# A Monte Carlo Method for Determining the Response Relationship between Two Commonly Used Detectors to Indirectly Measure Alpha Particle Radiation Activity

**DOI:** 10.3390/molecules24183397

**Published:** 2019-09-19

**Authors:** Christopher J. Tichacek, Mikalai M. Budzevich, Thaddeus J. Wadas, David L. Morse, Eduardo G. Moros

**Affiliations:** 1 Department of Radiation Oncology, H. Lee Moffitt Cancer Center & Research Institute, Tampa, FL 33612, USA; Christopher.Tichacek@moffitt.org; 2 Department of Cancer Physiology, H. Lee Moffitt Cancer Center & Research Institute, Tampa, FL 33612, USA; 3 Department of Physics, University of South Florida, Tampa, FL 33620, USA; 4 Small Animal Imaging Laboratory, H. Lee Moffitt Cancer Center & Research Institute, Tampa, FL 33612, USA; Mikalai.Budzevich@moffitt.org; 5 Department of Cancer Biology, Wake Forest University Health Sciences, Winston-Salem, NC 27157, USA; twadas@wakehealth.edu; 6 Department of Oncologic Sciences, University of South Florida, Tampa, FL 33612, USA

**Keywords:** alpha particle therapy, Actinium-225, Monte Carlo, radiation detection

## Abstract

Using targeted ligands to deliver alpha-emitting radionuclides directly to tumor cells has become a promising therapeutic strategy. To calculate the radiation dose to patients, activities of parent and daughter radionuclides must be measured. Scintillation detectors can be used to quantify these activities; however, activities found in pre-clinical and clinical studies can exceed their optimal performance range. Therefore, a method of correcting scintillation detector measurements at higher activities was developed using Monte Carlo modeling. Because there are currently no National Institute of Standards and Technology traceable Actinium-225 (^225^Ac) standards available, a well-type ionization chamber was used to measure 70.3 ± 7.0, 144.3 ± 14.4, 222.0 ± 22.2, 299.7 ± 30.0, 370.0 ± 37.0, and 447.7 ± 44.7 kBq samples of ^225^Ac obtained from Oak Ridge National Lab. Samples were then placed in a well-type NaI(Tl) scintillation detector and spectra were obtained. Alpha particle activity for each species was calculated using gamma abundance per alpha decay. MCNP6 Monte Carlo software was used to simulate the 4π-geometry of the NaI(Tl) detector. Using the ionization chamber reading as activity input to the Monte Carlo model, spectra were obtained and compared to NaI(Tl) spectra. Successive simulations of different activities were run until a spectrum minimizing the mean percent difference between the two was identified. This was repeated for each sample activity. Ionization chamber calibration measurements showed increase in error from 3% to 10% as activities decreased, resulting from decreasing detection efficiency. Measurements of ^225^Ac using both detector types agreed within 7% of Oak Ridge stated activities. Simulated Monte Carlo spectra of ^225^Ac were successfully generated. Activities obtained from these spectra differed with ionization chamber readings up to 156% at 147.7 kBq. Simulated spectra were then adjusted to correct NaI(Tl) measurements to be within 1%. These were compared to ionization chamber readings and a response relationship was determined between the two instruments. Measurements of ^225^Ac and daughter activity were conducted using a NaI(Tl) scintillation detector calibrated for energy and efficiency and an ionization chamber calibrated for efficiency using a surrogate calibration reference. Corrections provided by Monte Carlo modeling improve the accuracy of activity quantification for alpha-particle emitting radiopharmaceuticals in pre-clinical and clinical studies.

## 1. Introduction

The use of targeted ligands to deliver alpha particles directly to cancer cells has become a promising strategy to treat tumors [1,2,3,4]. This is because of their short path length of 40–80 µm and high linear energy transfer (LET~100 keV/µm) [5]. These two properties make alpha particles highly cytotoxic to targeted cells, with little damage to surrounding normal cells.

Owing to the large amount of energy deposited by alpha particles, it is important to quantify the radioactivity of the targeted radiopharmaceutical initially injected and its subsequent biodistribution, pharmacokinetics, and radiation dose deposited in various tissues. As a result of its short path length, the direct measurement of alpha particles’ activity in tissues is not possible in most, if not all, preclinical scenarios. If a parent metastable radioisotope also emits gamma rays, indirect methods of detection such as gamma spectroscopy can be used to estimate activity using scintillation detectors and well-type ion chambers that are commonly available in research laboratories and nuclear medicine clinics.

Actinium-225 (^225^Ac) was chosen as the radionuclide for alpha-particle therapy (TAT) targeted to the melanocortin 1 receptor for treatment of metastatic uveal melanoma [6], the prostate-specific membrane antigen (PSMA) for metastatic prostate cancer [1], and CD33 for acute myeloid leukemia [7]. Clinical studies have been carried out for the PSMA and CD33 TATs and others in pre-clinical development will soon go to clinical trials [6]

**Figure 1:**
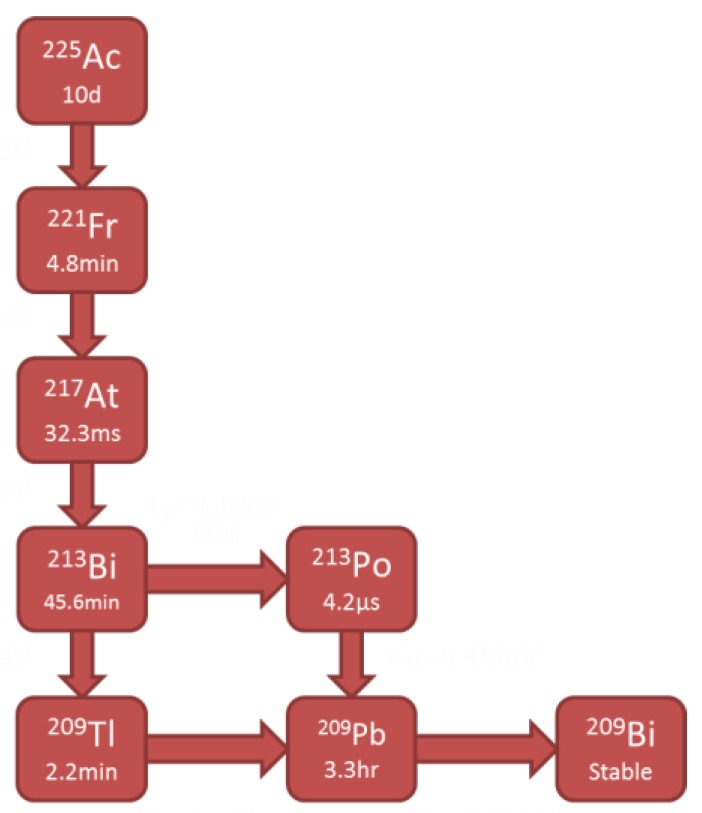
Decay chain of Actinium-225 (^225^Ac).

^225^Ac has a 10-day half-life and decays via six daughter radionuclides resulting in a net of four alpha particles per Actinium disintegration (Figure 1.) [8,9]. ^225^Ac and two of its daughters, ^221^Fr and ^213^Bi, also decay with accompanying isomeric gamma photons. Using gamma detection systems, such as ion chambers or scintillation detectors, it is possible to indirectly determine the alpha activity by gamma ray abundance per decay conversions.

In the clinic, ion chambers are readily available and their use is the standard of practice for checking activities for diagnostic and therapeutic agents. An ion chamber reading does not discriminate the collected charge between the parent and daughter radionuclides because it does not provide energy identification. In these situations, gamma spectroscopy using scintillation or semiconductor detectors, such as a sodium iodide doped with thallium scintillation detector [NaI(Tl)] or a high purity germanium (HPGe) detector, can be used. With this approach, it is possible to determine the activity of each gamma emitting daughter, which gives more information on how the decay species behave.

While HPGe seems to be the attractive option owing to its superior energy resolution, its performance suffers as a result of its low detection efficiency, cost, and requirement of sophisticated cooling systems. Despite the advantages of the NaI(Tl), dead time losses at higher activities and poor energy resolution may also provide incorrect activity measurements, leading to underestimation or overestimation of radiation dose [10].

In pre-clinical and clinical biodistribution studies, activity measurements of collected blood and tissues are used to calculate pharmacokinetics and radiation dosimetry. Therefore, accurate measurements for ^225^Ac and daughters are needed. Hence, scintillation detector measurements are needed to generate these spectra. Alpha radiation dosimetry is performed using the methods recommended by the Committee on Medical Internal Radiation Dose [11]. It is essential that these measurements are accurate because the dosimetry estimates are then extrapolated to human estimations and, therefore, serve as the fundamental basis for all patient safety measures.

Scintillation detectors are calibrated by analyzing three aspects of the detector: energy, resolution, and efficiency [12]. Once the detector is fully calibrated, it can be used to perform gamma spectroscopic measurements in order to determine the activity of radioactive samples [13,14,15,16,17,18]. Ion chambers are calibrated by measuring a sample of the radiopharmaceutical in question with known activity, correcting for decay, and applying a calibration factor. The American National Standards Institute (ANSI) recommends that the applied calibration factor adjusts the measurement to within ±10% of the known activity [19]. Although reference standards have been developed for isotopes used in internal radiotherapy, for example, ^177^Lu [20] ^225^Ac standards are currently under development by NIST and are not yet available. For this study, ^225^Ac provided by Oak Ridge National Lab was used as a cross-reference source.

While there are evident limitations in making clinical predictions when translating a radiopharmaceutical from mice to humans, it is important to minimize these limitations that are the result of instrumentation. Herein, a threshold is identified above which the scintillation detector cannot accurately measure the activities of ^225^Ac and daughters. An activity response relationship between the ion chamber and scintillation detector measurements is reported and used in a method to improve activity determination above the threshold by correcting scintillation detector measurements via Monte Carlo simulations. These corrections improve the dosimetry of pre-clinical work and ultimately facilitate the translation of the new radiopharmaceutical to the human clinic. 

## 2. Results

**Figure 2:**
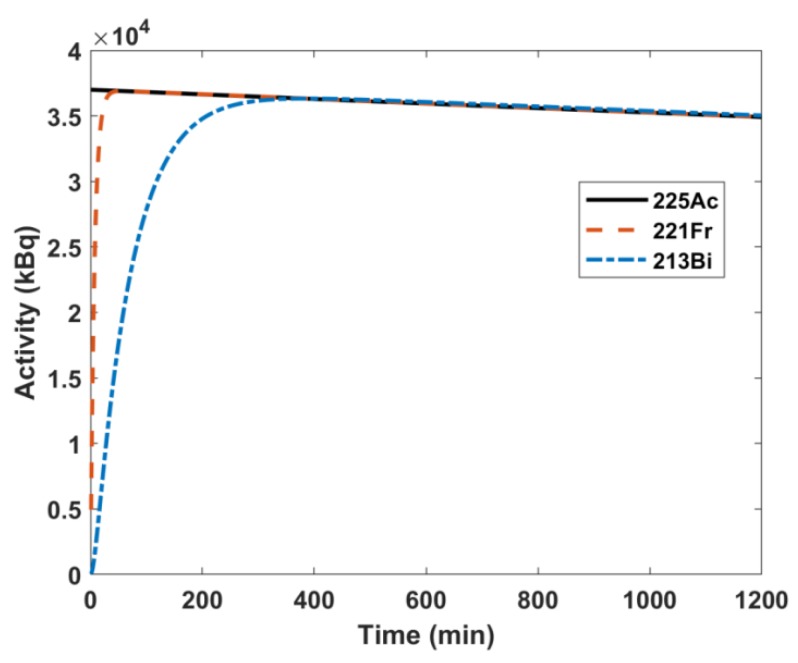
Solutions to system of decay ordinary differential equations of for an initial activity of 3.7 × 10^4^ kBq of ^225^Ac.

The system of ordinary differential equations describing the ^225^Ac decay chain was solved to determine the daughters’ activities as a function of time; these results are plotted in Figure 2 for ^225^Ac, ^221^Fr, and ^213^Bi. For an initial activity of 3.7 × 10^4^ kBq of ^225^Ac, ^221^Fr reaches secular equilibrium with ^225^Ac in 55 minutes, while ^213^Bi takes 380 minutes. Therefore, after 380 minutes (6.37 h), the parent and all daughter nuclides are in secular equilibrium.

**Figure 3:**
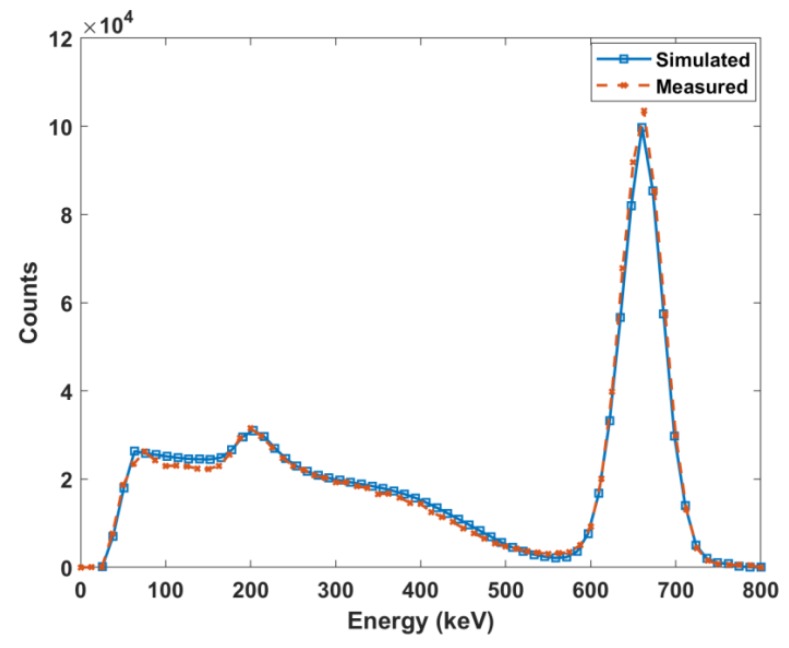
Comparison of measured and simulated spectra of ^137^Cs.

To account for the statistical variance of physical spectra, a Gaussian energy broadening function was integrated with the ideal Monte Carlo simulation model. The parameters of this function were determined by nonlinear fitting of the measured FWHM versus energy. These parameters were determined to be a = 0.005616, b = 0.0521, and c = 2.027 with an R-squared value of 0.9984. This allowed the benchmark validation of the Monte Carlo model using the ^137^Cs standard source (Figure 3). Simulating 155.4 kBq, the known source activity, showed a 1.02 mean percent difference at analogous bins between measured and simulated spectra. 

**Figure 4:**
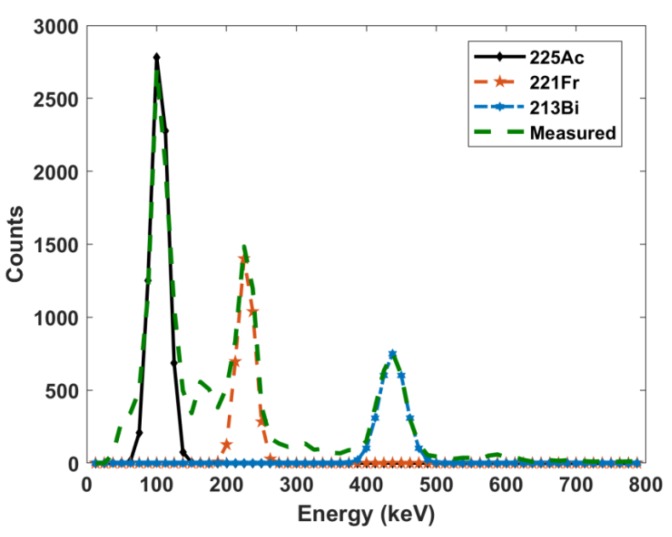
NaI(Tl) measured gamma spectrum with Gaussian fitting.

The measured NaI(Tl) gamma spectrum and Gaussian peak fitting of ^225^Ac and its daughters can be seen in Figure 4. The two source method was used to determine the dead time of the scintillation detector to characterize the saturation of the signal with increasing activity. The value was determined to be 4.6 × 10^−5^ s^-1^. The dead time value was incorporated into the gamma spectrum fitting method that was used to determine the scintillation detector activities of ^225^Ac, ^221^Fr, and ^213^Bi, and these values were compared to the ion chamber measurements (Table 1). All samples were in secular equilibrium during measurement, as shown in Figure 2. The ion chamber manufacturer error is reported as 3%. Measurements using NIST-traceable sources within the activity range of 5.705 to 39.257 MBq had 3% uncertainties. However, when measuring NIST-traceable sources at the lower activities of 158.73 and 391.09 kBq, the uncertainties were 9.7% and 9.6%, respectively.

**Figure 5:**
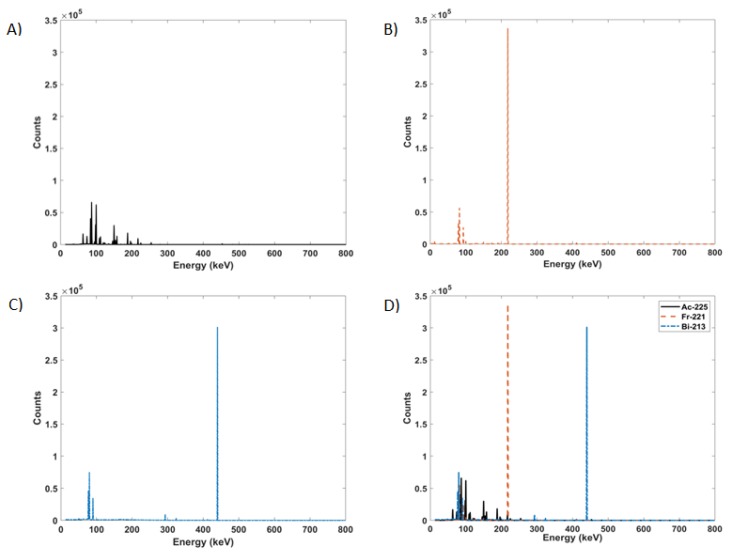
High definition Monte Carlo simulated gamma spectra of (**A**) ^225^Ac, (**B**) ^221^Fr, (**C**) ^213^Bi, and (**D**) all three superimposed.

The spectra acquired by the scintillation detector (Figure 4) have overlapping multiple peaks that are further masked by the poor resolution of the detector. Monte Carlo simulations with 1 keV resolution were run for the parent and gamma emitting daughter radioisotopes. As observed in Figure 5, ^225^Ac, ^221^Fr, and ^213^Bi all exhibit many peaks in the 90 to 200 keV range. Monte Carlo simulations allowed for the accounting of contributions from each individual radioisotope to the overlapping gamma spectra.

**Figure 6:**
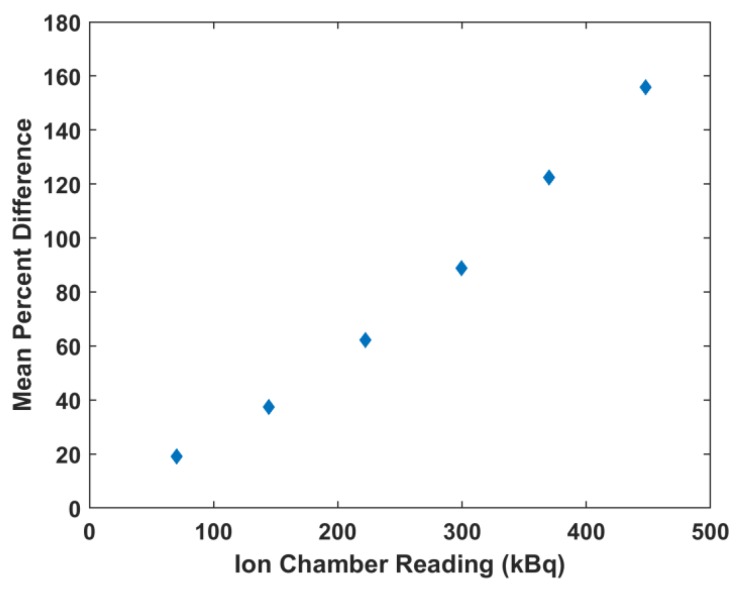
The mean percent difference between measured spectra and simulated spectra using the ion chamber reading measurement as input for the Monte Carlo model.

**Figure 7:**
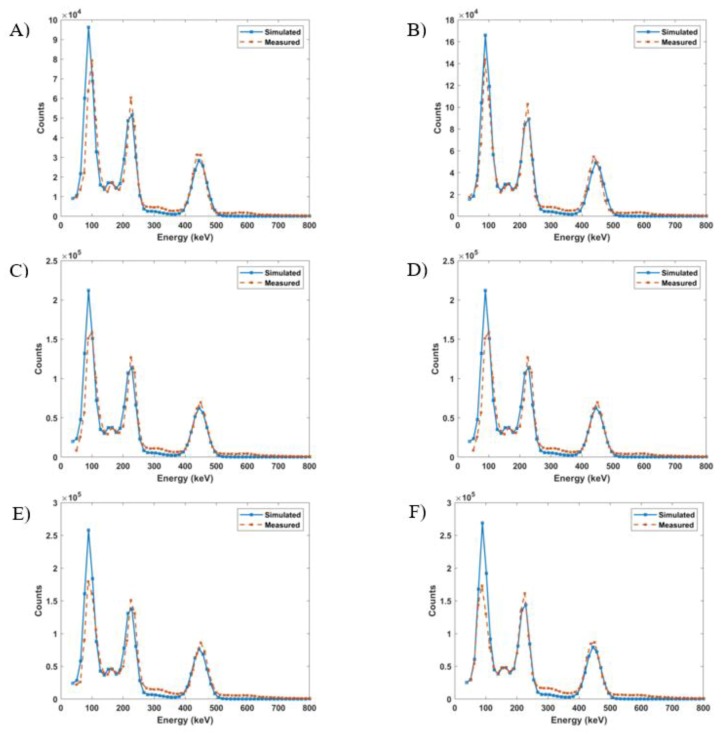
Measured vs. simulated ^225^Ac spectra for (**A**) 70.3 kBq (0.17%), (**B**) 144.3 kBq (0.5%), (**C**) 222.0 kBq (0.19%), (**D**) 299.7 kBq (0.99%), (**E**) 370.0 kBq (0.11%), and (**F**) 447.7 kBq (0.02%). The values in parentheses are the mean percent differences between the two spectra.

**Figure 8:**
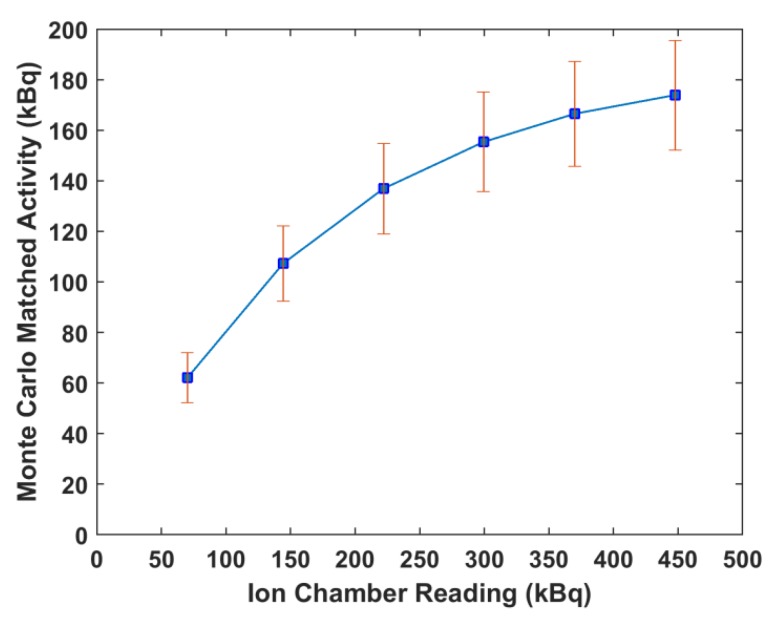
Activity response relationship between ion chamber reading and Monte Carlo-NaI(Tl) corrected spectra activity.

Monte Carlo simulations were run using the bin width of the NaI(Tl) detector and ion chamber readings. The resulting spectra were compared to the corresponding scintillation detector spectra and it was observed that as the readings increased, the percent difference between the measured and simulated spectra increased. The percent differences ranged from 19% at 70.3 kBq up to 156% at 447.7 kBq. This is illustrated in Figure 6. The readings that minimized the mean percent differences were used as the equilibrium input activities in the Monte Carlo simulations. Figure 7 shows the matching of simulated spectra to measured spectra for all six of the samples of different quantities. This procedure was repeated for all ion chamber readings. Figure 8 shows the ion chamber reading and the simulated activity that matches to within 1% of the NaI(Tl) spectra for all of the samples. At low readings, the measured and simulated quantities agree reasonably well: 70.3 kBq measured in the ion chamber matched with 62.9 kBq equilibrium Monte Carlo activity and 144.3 kBq measured in the ion chamber matched with 107.3 kBq Monte Carlo activity. For larger ion chamber readings, the matched response began to plateau.

## 3. Discussion

The purpose of this work is to develop a method to determine the activities of ^225^Ac and daughters in samples using standard clinical instruments. This is important for the determination of blood clearance pharmacokinetics and tissue biodistribution, both of which are used for radiation dosimetry calculations. In this work, an activity response relationship is determined between two radiation detectors commonly used for pre-clinical and clinical applications, a well-type NaI(Tl) scintillation detector and an ion chamber, respectively, where ^225^Ac activities between the range of 70 to 450 kBq are not accurately measured by the scintillation detector. This relationship was used to inform Monte Carlo simulations that corrected scintillation detector measurements for accurate readings within this range. Although the error of the ion chamber measurements increased to ~10% within this activity range, compared with errors of 3% observed when measuring activities as low as 6 MBq, the error within this range for the scintillation detector as compared with the benchmarked Monte Carlo simulations ranged from 20% to 160% (Figure 6).

Several preclinical studies have reported using a NaI(Tl) gamma counter to measure ^225^Ac injection and biodistribution activities [21,22,23,24,25]. Ion chambers are commonly used in nuclear medicine departments to prepare radiopharmaceuticals for patient injection. Measuring the activity of radioisotopes with complicated decay pathways, such as in the case of ^225^Ac, the readings are not only the result of the parent’s activity, but also from the contributions from the daughters’ activities. From the solution of the system of decay equations, it was determined that ^225^Ac and its daughters reached secular equilibrium in less than seven hours. While the daughters emit alpha particles and/or beta particles, the ranges of these particles are not penetrative enough to enter the ion chamber. Therefore, the ion chamber reading is a result of the gamma rays emitted from ^225^Ac, ^221^Fr, and ^213^Bi. For example, a recent human ^225^Ac therapy study reported injection activities for ^225^Ac, but it was not clear if the reported values were from bulk measurements or if spectra were acquired and only ^225^Ac emissions were considered [1].

Several groups have built Monte Carlo models of NaI(Tl) detectors to simulate response functions based on standard benchmark simulations and measurements of ^137^Cs without description of further extensions of their models [26,27,28,29,30,31]. Herein, this procedure was extended to include the detector response for ^225^Ac and its daughters. Implementation of the adjustment coefficients obtained by the nonlinear fitting into the Monte Carlo model adequately described the Gaussian spread of the detector pulses and was evident in benchmark testing. For all simulations, it was assumed that ^225^Ac, ^221^Fr, and ^213^Bi were in secular equilibrium and thus had equal activities. The high-definition Monte Carlo simulations showed that both ^221^Fr and ^213^Bi exhibit peaks in the energy range of ^225^Ac gamma pulse height distribution. Because of the 12.5 keV energy resolution of the NaI(Tl) detector, these peaks are summed to form a larger composite photopeak resulting in greater net counts, and thus over-calculated activities.

Gamma spectroscopic measurements were performed using the NaI(Tl) scintillation detector. All samples were measured after the calculated time to reach secular equilibrium, but the resulting activities were not equal. Further, the sum of activities for all three species greatly overestimated the ion chamber readings. Although the activities determined for the three isotopes should theoretically be identical, discrepancies are the result of the low energy bin resolution of the detector and the dead time count saturation. Using the measured ion chamber readings as input to the Monte Carlo simulations (Figure 6) largely overestimated the measured NaI(Tl) spectrum.

After minimizing the difference between each sample spectra and the Monte Carlo output, an activity response function relationship was determined for the two detectors (Figure 8). Both the Monte Carlo activity and the ion chamber reading represent the sum of the activities of ^225^Ac, ^221^Fr, and ^213^Bi. The plot shows a curve with a decreasing positive slope as the ion chamber measurements increased beyond 148 kBq. This is a result of the NaI(Tl) dead time losses. As the activity of a sample increases, more pulses are lost, leading to errors in counting. The discrepancy between the low energy measured and simulated peaks in Figure 7 is also a result of dead time effects. The ratio of simulated to measured peaks increases with increasing activity because the Monte Carlo model does not account for dead time. Further, the disagreement in the simulated and measured spectra in the low count region arises from the fact that the model represents an idealized detector and does not account for the metallic impurities of a real detector.

Standard NIST-traceable samples of ^225^Ac are currently unavailable, so ^225^Ac obtained from Oak Ridge was used as a surrogate calibration reference. These samples did not come with uncertainty estimations. As measurements using the recommended dial number agreed with the Oak Ridge surrogate reference samples to less than 10%, the readings of the ionization chamber were considered reliable for the study carried out in this work. The scintillation detector’s measured energy resolution and detection efficiency over the energy range of ^225^Ac gamma emissions (40-662 keV) agreed with other published studies and reports [28,32,33,34]. Consequently, this detector was also considered to be calibrated in efficiency and energy for ^225^Ac analysis. The calibration sources used were all less than 185 kBq. When calibrating the ion chamber with sources with activity above 5.71 MBq, the error was less than 3%. When testing with sources in the lower activity range, error in the measurements increased to 10%, which is still within ANSI recommended tolerance. The measurement results showed that the responses from these two detectors do not correlate well in the activity range of 70 to 450 kBq, even though both detectors were calibrated for efficiency and energy resolution according to their respective recommended methods. The Monte Carlo model developed herein corrects NaI(Tl) measurements, allowing for a more accurate estimate of ^225^Ac and daughter activities over a range of activities useful for both pre-clinical and clinical use. 

## 4. Materials and Methods

### 4.1. Mathematical Model

**Figure 9:**
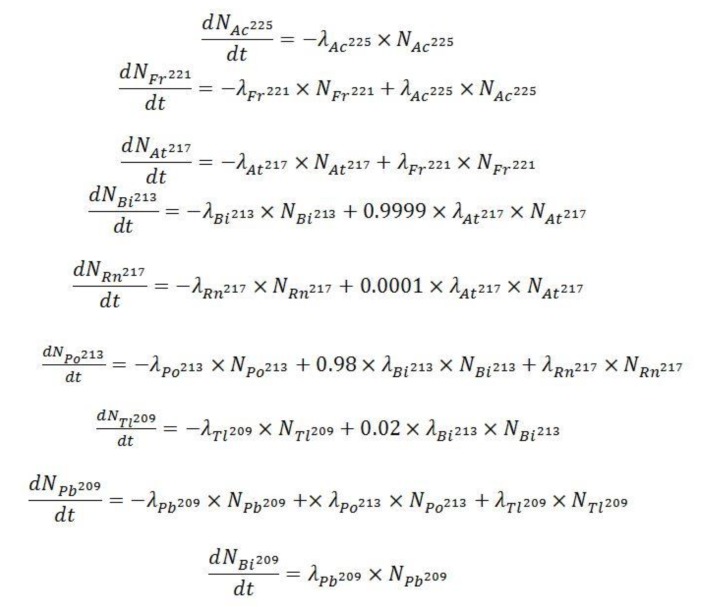
System of equations describing the decay of ^225^Ac to the very long-lived ^209^Bi.

The ^225^Ac decay chain can be characterized by a system of ordinary differential equations that describe the activities and abundances of each species as a function of time (Figure 9) [35].

In Figure 9, $N_{x}$ and $\lambda_{x}$ denote the number of atoms of radionuclide and the decay constant of radionuclide *X*, respectively. These equations were simultaneously solved in Mathematica with the initial (time zero) ^225^Ac activity set to 3.7 x 10^4^ kBq and all daughters’ activities set to 0 kBq to determine the times at which daughter products reach equilibrium with the parent. 

### 4.2. Monte Carlo Model

**Figure 10:**
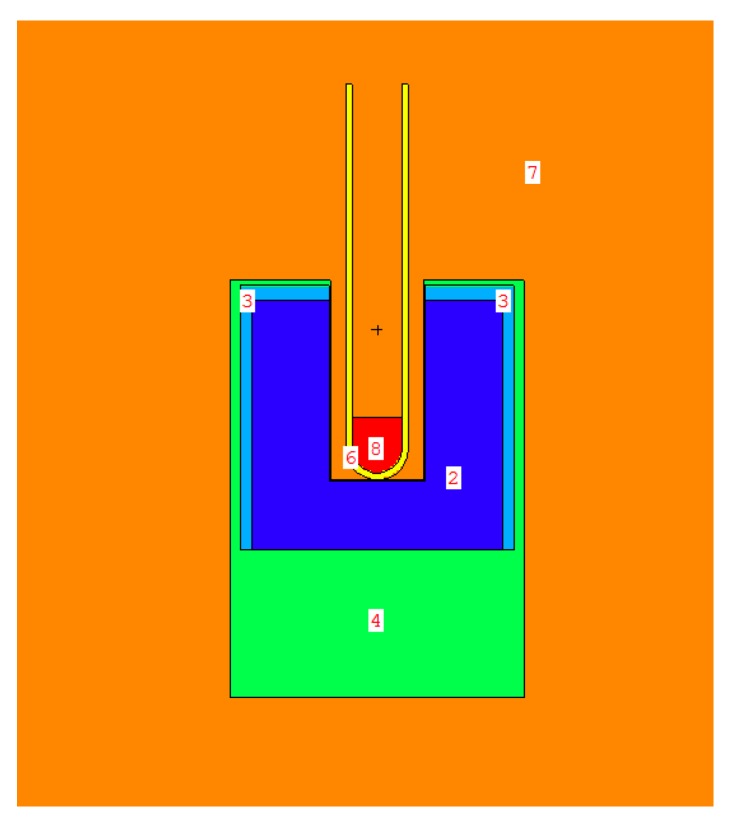
Geometry of the Monte Carlo model wipe test scintillator detector. Image generated using the Visual Editor software for MCNP. 2: NaI; 3: MgO; 4: Al; 6: polyethylene; 7: air; 8: H_2_0 with ^225^Ac, ^221^Fr, ^213^Bi source distributions.

The Monte Carlo N-Particle version 6.1 (MCNP6.1) package was used to simulate the pulse height gamma distribution from ^225^Ac [36]. A computational model of the 2” × 2” NaI(Tl) 4π detector was built using the material compositions and dimensions given in the manufacturer’s manual, as illustrated in Figure 10 [32]. 

A standard 5 mL polyethylene test tube filled with 1 mL of water was placed in the well of the detector. The source was defined to be uniformly distributed in the 1 mL water volume. All 75 photon emissions from ^225^Ac, ^221^Fr, and ^213^Bi were defined by energy and abundance [9]. The photomultiplier tube was modeled as a 30 mm diameter aluminum cylinder to account for backscatter [26].

Photon transport was conducted using the mcplib84 photoatomic data library and detailed photon physics interactions [37]. These include Thomson scattering, Compton scattering, and photoelectric absorption with the creation of fluorescence photons or Auger electrons. Full photon and electron simulations were performed and spectra were compared to those obtained from only photon transport. Photon and electron cell importances were set to 1 for all cells in the geometry. A mean percent difference of less than 1% between photon/electron transport and photon only transport was observed in benchmark generated spectra of ^137^Cs. There were also less than 1% differences in the Compton edge and Compton regions. Therefore, the computationally expensive tracking of electrons was omitted in subsequent simulations and electron cell importances were set to 0. In this case, the electrons generated are assumed to deposit their energy locally. Any electron induced bremsstrahlung photons produced are accounted for in the thick-target bremsstrahlung model [38]. This model skips the electron tracking step and transports the generated photon in the direction of the parent electron.

The pulse height distribution tally (F8) was used to calculate the deposited energy in the NaI(Tl) crystal. This tally accumulates the kinetic energy lost by local photon-induced secondary electrons, per history, in a specified volume. The tally was divided into 64 bins, each with a 12.5 keV width to match the energy resolution of the detector. To relate the simulated response to activity, values for the number of gamma emissions per disintegration for each isotope, scaled by the activity, were placed on the source probability card in the source definition. The resulting tallies were then multiplied by the sum of these values, the live acquisition time, and an activity conversion factor containing photon abundance per decay. For each simulation, 2 x 10^8^ histories were run to achieve less than 1% error under each photopeak.

MCNP6.1 models an ideal detector and thus does not take into account the Gaussian shape of experimental NaI(Tl) energy resolution resulting from intrinsic light collection inefficiency, statistical fluctuation in charge multiplication in the photomultiplier tube, and electronic noise [39]. To account for the statistical variance of physical spectra, a Gaussian energy broadening function (MCNP6.1 FT8 GEB) was applied to the simulated pulse height distribution. The GEB function was obtained experimentally from measurements of the detector’s specific full width at half maximum (FWHM) from a set of isotopes of differing energies. Spectra from five isotopes were measured and the FWHM of each peak was calculated. The isotopes used and their physical characteristics are listed in Table 2.

A curve of FWHM as a function of energy was fit with the following nonlinear function shown in Equation (1) [36]:

(1) $$FWHM=a+b\sqrt{E+cE^{2}},$$

where E is the incident energy of the incident photon in keV; and a, b, and c are parameters that were determined by least squares fitting and input in the FT8 command.

### 4.3. Monte Carlo Simulations

There are no standard NIST-traceable calibration sources for ^225^Ac to validate the detector model. Instead, the spectrum of a NIST-traceable 155.4 kBq ^137^Cs (662 keV photopeak) source was measured in the NaI(Tl) detector and compared to a simulated spectrum of the same activity for validation.

After the model was validated, a high definition version of the F8 pulse height tally (1 keV bin width) was simulated with only ^225^Ac defined as the source. This was followed by separate high definition simulations of ^221^Fr and ^213^Bi. These simulations were done to see what radiations contribute to each peak, which are masked by the poor energy resolution of the NaI(Tl) detector. 

### 4.4. Experimental Measurements

The ^225^Ac used in this work was obtained from Oak Ridge National Laboratory (ORNL). The Atomlab^TM^ Wipe Test Counter (Biodex Medical Systems, Inc., Shirley, New York, USA) was used to perform gamma spectroscopy measurements with ^225^Ac. The wipe test counter is a 2” × 2” NaI(Tl) well-type scintillation detector with 1.2 cm of lead shielding. The multichannel analyzer associated with the wipe test NaI(Tl) detector has 64 12.5 keV channels ranging from 0 to 800 keV. Spectra obtained with the NaI(Tl) detector for 30 s acquisition time and full energy window to include gamma counts from ^225^Ac, with composite peak at 99.8 keV, and from its two gamma-emitting daughters, ^221^Fr (T_1/2_ = 4.9 min) with a peak at 218.1 keV and ^213^Bi (T_1/2_ = 46 min) with a peak at 440.5 keV. Background spectrum measurements were performed and subtracted from all obtained ^225^Ac spectra. The net background readings for the NaI(Tl) detector were in the range of 100 to 200 net counts per minute. The net counts for an ^225^Ac sample of 45 kBq activity was 444,000 per minute.

Dead time measurements and calculations were performed according to the two-source method [10]. Two different sources of ^22^Na with different activities were each measured individually and then in combination.

In-house software was developed using MATLAB to calculate the net number of gamma counts, N, by fitting each peak with a Gaussian fit and integrating. Alpha particle activity for each species was calculated using factors for gamma abundance (Y) per alpha decay: ^225^Ac (1%), ^221^Fr (11.4%), ^213^Bi (25.9%) [9].

The detector efficiency, ε, and energy calibration for the NaI(Tl) detector were determined by performing measurements of NIST-traceable ^137^Cs,^133^Ba, and ^57^Co sources. These radionuclides have numerous photopeaks covering the gamma energy spectrum of ^225^Ac. The activity is then given by the following Equation (2):

(2) $$A=\;\frac{N}{t*\varepsilon *Y},$$

where t is live time. The activity of ^225^Ac determined using this approach was compared to a decay corrected sample of known activity from ORNL. A factor of 0.082 was applied to Equation (2) to obtain ^225^Ac activity values to within 5% of the ORNL specified ^225^Ac activity. The uncertainty in the NaI(Tl)-determined activity was calculated by propagating individual uncertainties in the net number of counts and the efficiency measurements, as is typically done in nuclear counting measurements. 

The Atomlab^TM^ 500 Dose Calibrator is a well-type pressured ion chamber filled with argon gas that is commonly used in the clinic. This instrument was calibrated for energy and efficiency with the following NIST-traceable sources: ^137^Cs (6.63 MBq), ^57^Co (39.26 MBq), and ^133^Ba (5.71 MBq) by the vendor. Lower activity NIST-traceable sources provided by Eckert & Ziegler were also used: ^137^Cs with 158.73 kBq and ^57^Co with 391.09 kBq activities. No significant variation in the calibration coefficient as a function of photon energy was observed. An ion chamber linearity test was performed with ^18^F in the activity range of 1.2 GBq to 266.0 kBq and a variance of 1.4% was observed. The dial number was set at 38.2 for ^225^Ac, as indicated by the vendor and confirmed by our comparison with two decay corrected samples of different activities obtained from Oak Ridge. Measurements of these samples in the ion chamber agreed with the Oak Ridge measurements to within 7%. In order to determine the relationship between this ion chamber and the NaI(Tl) detector, six different activities of ^225^Ac samples were measured using the ion chamber: 70.3 ± 7.0, 144.3 ± 14.4, 222.0 ± 22.2, 299.7 ± 30.0, 370.0 ± 37.0, and 447.7 ± 44.7 kBq. Each sample was measured and placed in the NaI(Tl) detector, and a spectrum was obtained and compared. Using the activity reading from the ion chamber as the initial parent–daughter equilibrium activity input for the Monte Carlo model, simulated spectra were calculated and compared to the measured spectra from the NaI(Tl) detector. Mean absolute percent differences at analogous bins between the experimental and simulated spectra were calculated. Using the ion chamber measured activity as input to the Monte Carlo simulations underestimated the readings from the NaI(Tl), so several successive runs were completed using different activities as input in symmetrical range around the initial point in order to match the spectra. The percent differences were plotted against input activity and fitted with a linear model. The x-intercept was found in order to determine the minimum mean percent difference between the measured and simulated spectra. This process was repeated for each sample and an activity response function relationship was determined between the ion chamber and the NaI(Tl) detector. The uncertainty in the activity relationship determined using this methodology was analyzed by adding the uncertainties in the NaI(Tl) measurements, $\sigma_{NaI}^{2}$; the ion chamber measurements, $\sigma_{IC}^{2}$; and the Monte Carlo calculations, $\sigma_{MC}^{2}$, in quadrature (Equation (3)):

(3) $$\sigma_{A}=\sqrt{\sigma_{NaI}^{2}+\sigma_{IC}^{2}+\sigma_{MC}^{2}}.$$

## 5. Conclusions

In this work, the ^225^Ac activity response function between two commonly used instruments was investigated, a range of activities (70 to 450 kBq) was identified, and a method was developed using Monte Carlo simulations to correct scintillation detector measurements within this range. The scintillation detector was calibrated in energy and efficiency using NIST-traceable sources covering a broad energy range, while the ion chamber was calibrated in efficiency using a surrogate calibration reference. The error of ion chamber measurements increased with decreasing activity, but remained within the ANSI recommended value of 10%. The NaI(Tl) performed poorly from 70 to 450 kBq. Therefore, a Monte Carlo model was built to correct the NaI(Tl) measurements, thereby providing improved accuracy. The corrections provided by the Monte Carlo model will provide a useful tool to make corrections to the activity values measured by the well-type NaI(Tl) detector. This allows for the better quantification of alpha-particle emitting radiopharmaceuticals and daughter byproducts in pre-clinical and clinical studies. In this case, use of scintillation gamma spectroscopy will be the preferred method.

## Figures and Tables

**Table 1:** Ion chamber and NaI(Tl) measured activities. Single measurements were taken in each detector. Notice that as the ion chamber readings increase, the discrepancies with the NaI(Tl) increase. All values in kBq. Note that the uncertainties in the ion chamber readings are expressed as 10% in accordance with the calibration measurements in the activity range (above). The NaI(Tl) uncertainties are expressed as the propagation of uncertainties in all steps of the calculation. The difference between the ion chamber readings and the NaI(Tl) determined activity for ^225^Ac are indicated in the last column to the right.

Ion Chamber Reading	NaI(Tl) Determined Activities			
	^225^Ac	^221^Fr	^213^Bi	^225^Ac Percent Difference
70.3 ± 7.0	57.35 ± 7.57	66.97 ± 8.18	63.64 ± 7.98	20.23
144.3 ± 14.4	96.20 ± 9.81	115.07 ± 10.73	111.74 ± 10.57	40.00
222.0 ± 22.2	124.69 ± 11.17	152.81 ± 12.36	143.93 ± 12.00	56.14
299.7 ± 30.0	140.23 ± 11.84	177.97 ± 13.34	165.39 ± 12.86	72.50
370.0 ± 37.0	152.07 ± 12.33	198.32 ± 14.08	182.41 ± 13.51	83.49
447.7 ± 44.7	140.23 ± 11.84	211.64 ± 14.55	187.59 ± 13.70	104.59

**Table 2:** Physical characteristics of the radioactive sources used for NaI(Tl) detector energy response measurements [9].

Source	Half-Life	Energy (keV)
^129^I	1.6^7^ yr	39.5
^241^Am	432.6 yr	59.5
^57^Co	271.7 d	122.0
^68^Ga	67.7 m	511.0
^137^Cs	30.1 yr	661.6
